# Cutaneous Management after Extravasation of High-Concentrated Amino Acid Solution Administered for Renal Protection in PRRT

**DOI:** 10.3390/tomography8010029

**Published:** 2022-02-03

**Authors:** Chaninart Sakulpisuti, Wichana Chamroonrat, Supatporn Tepmongkol

**Affiliations:** 1 Division of Nuclear Medicine, Department of Diagnostic and Therapeutic Radiology, Faculty of Medicine Ramathibodi Hospital, Mahidol University, Bangkok 10400, Thailand; chaninart.sak@mahidol.ac.th; 2 Division of Nuclear Medicine, Department of Radiology, Faculty of Medicine, Chulalongkorn University, Bangkok 10330, Thailand; supatporn.t@chula.ac.th; 3 Chulalongkorn University Biomedical Imaging Group (CUBIG), Department of Radiology, Faculty of Medicine, Chulalongkorn University, Bangkok 10330, Thailand

**Keywords:** peptide receptor radionuclide therapy (PRRT), extravasation, amino acid, cutaneous lesion

## Abstract

High-concentrated amino acid solution is used to protect the kidneys during peptide receptor radionuclide therapy (PPRT) in patients with neuroendocrine tumors (NETs). Extravasation of the solution can cause cutaneous complications. In this study, we described a 66-year-old man with metastatic medullary thyroid cancer and a 32-year-old woman with metastatic pancreatic NET who developed cutaneous lesions caused by the extravasation of an amino acid solution (25 g of lysine and 25 g of arginine in 1 L of normal saline) during PRRT with [^177^Lu]Lu-DOTA-TATE. Both were treated conservatively, and these cutaneous lesions gradually improved. The patient with metastatic pancreatic NET rejected the amino acid infusion in subsequent cycles of PRRT and therefore received [^177^Lu]Lu-DOTA-TATE alone, and her serum creatinine level and estimated glomerular filtration rate (eGFR) remained normal for 2 months after the last treatment. These two cases revealed cutaneous complications resulting from high-concentrated amino acid solution during PRRT because of hyperosmolarity. Health care providers should be aware of this complication to ensure its prevention and appropriate management. Preserved renal function was demonstrated after [^177^Lu]Lu-DOTA-TATE treatment in the absence of the infusion of a high-concentrated amino acid solution. However, long-term follow-up of renal function is suggested.

## 1. Introduction

Peptide receptor radionuclide therapy (PRRT), consisting of a Lutetium-177 (Lu-177)– or Yttrium-90 (Y-90)–labeled somatostatin analog compound, is indicated for patients with positive somatostatin receptor (SSTR) type 2 expression or metastatic or inoperable neuroendocrine tumors (NETs) [1]. The kidneys are exposed to high levels of ionizing radiation when a radiolabeled somatostatin analog is excreted from the body. In proximal renal tubules, radiolabeled somatostatin analogs are reabsorbed by the megalin/cubilin receptor complex and degraded into metabolites, which are retained in the renal interstitium, causing the retention of radioactivity in the kidneys. Moreover, the kidneys all express subtypes of SSTR, resulting in the renal uptake of radiolabeled somatostatin analogs [2]. The standard treatment protocol requires the administration of an amino acid solution with the appropriate lysine and arginine concentrations for renal protection, which is achieved by decreasing resorption and thereby decreasing the radiation dose to the kidneys [1,3]. The compounded amino acid formula, consisting of 25 g of lysine and 25 g of arginine diluted in 1 L of normal saline, is preferred because it is more tolerable [3].

Cutaneous complications caused by extravasation of arginine and parenteral fluid containing amino acids have been described [4,5,6,7,8,9], but the use of high-concentrated amino acid solutions in patients undergoing PRRT is less known. We report two patients who developed cutaneous lesions during the infusion of a lysine and arginine amino acid solution.

## 2. Methods

### 2.1. Case 1

A 66-year-old man with medullary thyroid cancer and nodal metastasis in the neck and mediastinum underwent total thyroidectomy, central neck dissection, bilateral neck dissection, and median sternotomy with mediastinal node dissection, followed by three cycles of PRRT to treat partially unresected mediastinal lymphadenopathy. Subsequently, he was placed on active surveillance, and no additional treatment was prescribed.

**Figure 1:**
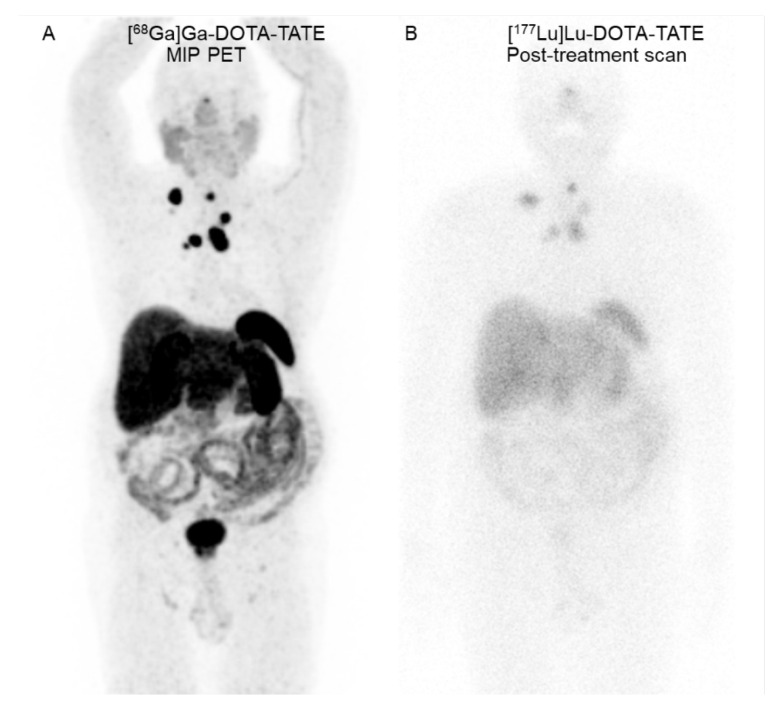
A 66-year-old man with metastatic medullary thyroid cancer. (**A**) Coronal maximum-intensity projection (MIP) [^68^Ga]Ga-DOTA-TATE PET image reveals SSTR-positive lesions in the residual tumors at the pre-tracheal region and neck, mediastinal, and right internal mammary metastatic lymph nodes. (**B**) Seven-day [^177^Lu]Lu-DOTA-TATE post-treatment scan reveals [^177^Lu]Lu-DOTA-TATE uptake in all known SSTR-positive residual tumors and nodal metastases.

After 4 years, local recurrence and the progression of nodal metastases were found. He underwent salvage neck dissection. Post-operative [^68^Ga]Ga-DOTA-TATE positron emission tomography/computed tomography (PET/CT) revealed multiple SSTR-positive residual tumors in the pre-tracheal region and nodal metastases in the bilateral supraclavicular, mediastinal, and right internal mammary regions (Figure 1). An additional three cycles of [^177^Lu]Lu-DOTA-TATE (7400 MBq per cycle) separated by 8-week intervals were completed.

The standard treatment protocol included pre-medication with dexamethasone and ondansetron and the administration of the high-concentrated amino acid solution for renal protection. The solution was infused over 4 h starting 30 min before the administration of [^177^Lu]Lu-DOTA-TATE via an infusion pump [^177^Lu]Lu-DOTA-TATE was infused through a different intravenous (IV) access point. His baseline serum creatinine level was 0.93 mg/dL (reference range, 0.73–1.18 mg/dL), and his estimated glomerular filtration rate (eGFR) according to the Chronic Kidney Disease-Epidemiology Collaboration (CKD-EPI) equation was 85.8 mL/min/1.73 m^2^.

**Figure 2:**
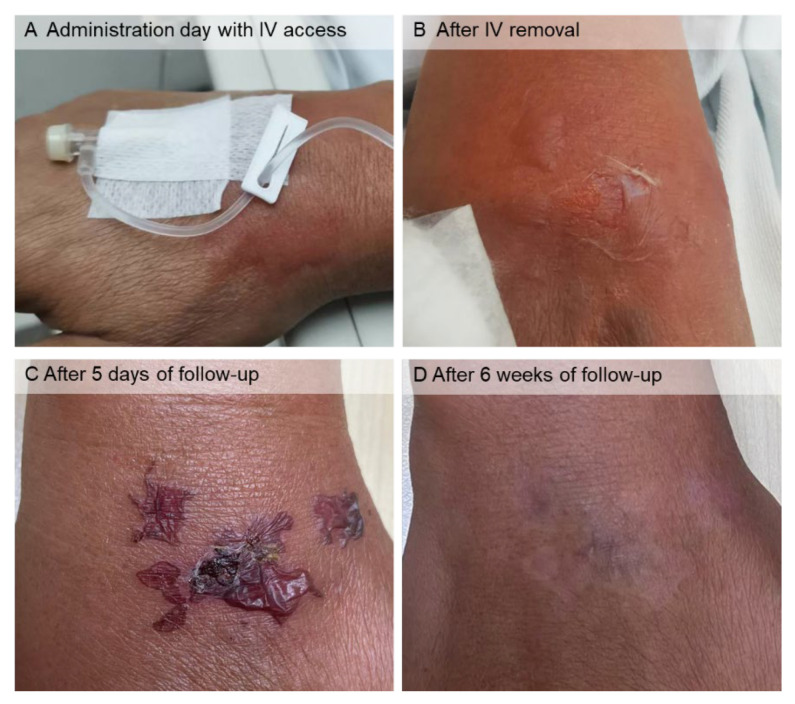
Cutaneous lesions in the right-hand dorsum after lysine and arginine solution extravasation. On the administration day with IV access ((**A**), dorsolateral view) and IV removal ((**B**), dorsal view), there were bullae, and the largest one ruptured, producing skin erosion. (**C**) After 5 days of follow-up, the bullae dried, and there was a crust in the skin erosion. (**D**) After 6 weeks of follow-up, the lesions were completely healed, leaving well-defined mixed hypo- and hyperpigmented patches.

The first cycle was completed without any adverse effects. During the second cycle, the patient complained of pain in his right hand toward the end of the amino acid infusion after toileting. Shortly afterward, bullae with skin erosion developed in the right-hand dorsum (Figure 2), the site of the lysine and arginine infusion. The remaining amino acid solution (less than 100 mL) was withdrawn and discarded. Skin dressing with cold compression was applied. Amino acid extravasation was presumably the cause. Saline dressing and aescin, and diethylamine salicylate gel-cream were advised, and the skin lesions were subsequently improved after this treatment (Figure 2). The third cycle with a similar high-concentrated amino acid infusion was administered without complications. One month after the third cycle, his serum creatinine level and eGFR (CKD-EPI) were 0.87 mg/dL and 90.6 mL/min/1.73 m^2^, respectively. Nine months after the third cycle, his serum creatinine level and eGFR (CKD-EPI) were 0.92 and 86.4 mL/min/1.73 m^2^, respectively.

### 2.2. Case 2

**Figure 3:**
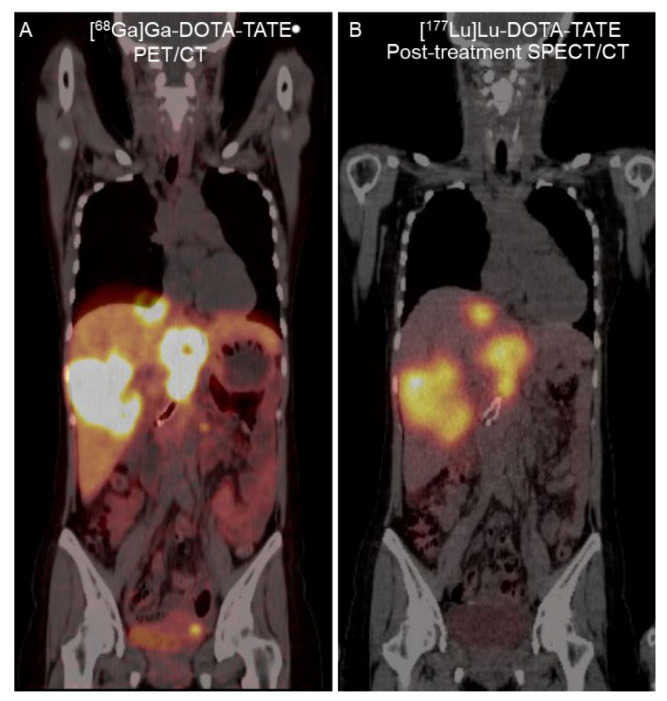
A 32-year-old woman with metastatic pancreatic NET. (**A**) Coronal fused [^68^Ga]Ga-DOTA-TATE PET/CT image revealed SSTR-positive liver metastasis. (**B**) Twenty-four-hour [^177^Lu]Lu-DOTA-TATE post-treatment fused single-photon emission computed tomography (SPECT)/CT image revealed similar [^177^Lu]Lu-DOTA-TATE uptake in the liver metastasis.

A 32-year-old woman with metastatic pancreatic NET (grade II, Ki-67 of 10%) presented with abdominal pain. She had undergone surgery of the pancreatic tail 5 years previously and received targeted therapy with everolimus, sunitinib, lanreotide, and temozolomide for 2 years. [^68^Ga]Ga-DOTA-TATE PET/CT revealed multiple SSTR-positive lesions in the bilateral hepatic lobes and intra-abdominal lymph nodes (Figure 3). Thus, she was referred to the Nuclear Medicine Department for PRRT. Four cycles of [^177^Lu]Lu-DOTA-TATE (7400 MBq per cycle) separated by 8-week intervals were proposed. Her baseline serum creatinine level was 0.43 mg/dL (reference range, 0.50–1.00 mg/dL), and her eGFR (CKD-EPI) was 135.01 mL/min/1.73 m^2^.

**Figure 4:**
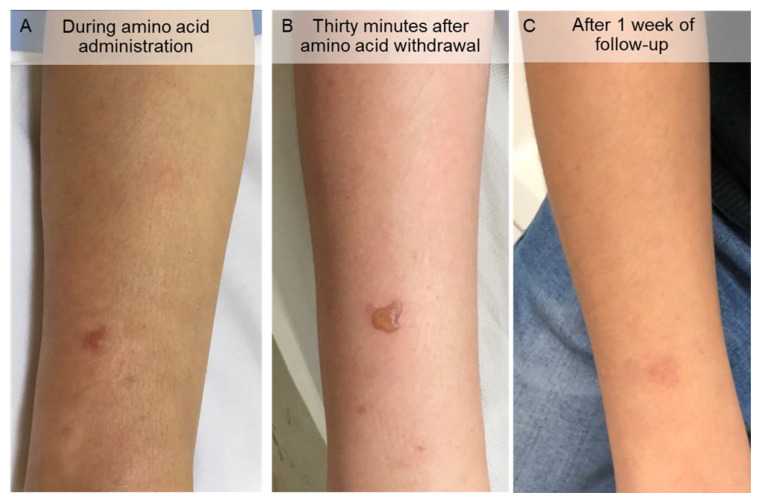
Cutaneous lesions on the right forearm after lysine and arginine solution extravasation. (**A**) During the amino acid infusion, bulla and wheals appeared on the forearm. (**B**) Thirty minutes after amino acid withdrawal, most of the wheals disappeared, although the bulla persisted. (**C**) After 1 week of follow-up, the bulla resolved, leaving a hyperpigmented area.

Similar to the first case, the patient received pre-medication with dexamethasone and ondansetron. Then, the high-concentrated amino acid solution was infused over 4 h starting 30 min before the administration of [^177^Lu]Lu-DOTA-TATE via an infusion pump. Three weeks after the first cycle, she experienced acute cholangitis and received an IV antibiotic. The second cycle was completed without any adverse effects. During the third cycle, she developed bullae and wheals in an area ranging from the right hand to the right forearm proximal to the site of the lysine and arginine infusion (Figure 4) in the right-hand dorsum. She had mild pain in the affected area. Amino acids were immediately withdrawn, and a dermatologist was consulted. After discussion with the dermatologist, amino acid extravasation was presumed to be the cause. A topical steroid was prescribed. The wheals gradually resolved approximately 15 min to 1 h after withdrawal, but the vesicles and blebs persisted for approximately a week (Figure 4).

During the fourth cycle, she refused amino acid pre-medication. Her serum creatinine level and eGFR (CKD-EPI) before this cycle were 0.53 mg/dL and 125.15 mL/min/1.73 m^2^, respectively. Her follow-up creatinine level and eGFR (CKD-EPI) 6 months after the fourth cycle were 0.44 mg/dL and 133.05 mL/min/1.73 m^2^, respectively.

After the fourth cycle, she was treated with octreotide. Because her abdominal pain persisted and she developed peritoneal metastasis, an additional two cycles of [^177^Lu]Lu-DOTA-TATE, each at a dose of 7400 MBq with an 8-week interval, were administered without a high-concentrated amino acid infusion. Two months after the sixth cycle, her serum creatinine level and eGFR (CKD-EPI) were 0.51 mg/dL and 125.86 mL/min/1.73 m^2^, respectively.

## 3. Discussion

The NETs are a heterogeneous group of malignancies originating from the diffuse neuroendocrine cell system and frequently overexpressing SSTR [3,10]. It is estimated that more than 12,000 people in the United States are diagnosed with a NET each year [11]. While they can arise in any organ of the body, the most common site is in the gastroenteropancreatic region accounting for 70% [10]. About 25% arise in the bronchopulmonary system, and less than 5% arise at other sites [1].

Surgical resection is considered the only curative option for NETs. For metastatic NETs, resection is currently recommended if curation can still be reached by resecting the primary tumor and all metastases or in symptomatic patients [12]. There are multiple systemic therapies for metastatic NETs, including somatostatin analogs, PRRT, targeted therapy, chemotherapy, and interferon-alpha [1,10,12].

PRRT is a molecular therapy using a radiolabeled-somatostatin analog compound. It is indicated for patients with positive expression of SSTR type 2 or metastatic or inoperable NETs [1]. After administration, radiolabeled-somatostatin analogs bind to SSTRs in the NETs and are internalized as per the normal receptor recycling dynamic, and the breakdown product of radiolabeled-somatostatin analogs are stored in lysosomes, thus enabling radiation dose delivery inside the tumor cells. This leads to radiation-induced DNA damage and cell death [13]. Beta-emitting radionuclides, Lu-177 or Y-90, are the most commonly used radionuclides [1].

Because of the high radiation dose to the kidneys during PRRT, a high-concentrated amino acid solution with appropriate lysine and arginine concentrations is recommended for renal protection [3]. The most common side effects of this solution are nausea and vomiting [1,3] though extravasation can occur and result in tissue damage.

The risks of extravasation depend on (1) mechanical factors (e.g., sizes of the vein and catheter, administration site, patient’s activity); (2) physiologic factors including clot formation above the cannulation site, thrombus or fibrin at the catheter tip, and lymphedema; and (3) pharmacologic factors such as pH, osmolarity, vasoconstriction, and cytotoxic potential [14]. The possible pathogenic mechanisms of tissue injury induced by lysine and arginine solution are acidic pH and hyperosmolarity. Extreme pH (both alkaline and acidic) and hyperosmolarity can irritate venous endothelium, and the vessel wall is susceptible to rupture, allowing the solution to extravasate and damage the surrounding tissue [14,15]. The degree of tissue injury is determined by the pharmacologic characteristics of the extravasating fluid and other factors, such as the concentration and amount of leakage, extravasation site, duration of tissue exposure, and underlying disease state [14]. Moreover, extravasation of hyperosmotic solution causes a fluid shift from inside the cell to the interstitial space resulting in swelling and increased local pressure, which can cause acute limb compartment syndrome [14].

Several case reports demonstrated skin lesions attributable to the extravasation of hyperosmotic solutions such as arginine monohydrochloride and parenteral fluid containing amino acids [4,5,6,7,8,9]. In severe cases, patients experienced skin necrosis and required surgical treatment [4,5,6,8]. Our two patients presented with cutaneous lesions, bullae, and wheals in particular, which were caused by the extravasation of a high-concentrated amino acid solution consisting of 25 g of lysine and 25 g of arginine during PRRT with [^177^Lu]Lu-DOTA-TATE. Because the infusions were immediately withdrawn, the skin damage was not permanent. The lesions were treated conservatively, and they gradually improved.

In the event where extravasation has occurred, the following general steps are recommended [16]: (1) stop administration of IV fluid immediately, (2) disconnect the IV tube from the cannula, (3) aspirate any extravasated solution from the cannula with a 10 to 20-mL syringe, (4) elevate the limb with the extravasation site, and (5) notify the attending physician. Cutaneous lesions such as erythema, wheals, and blebs or bullae caused by extravasation of high-concentrated amino acid solution administered in PRRT can be treated conservatively. Our patients were treated with saline-wound dressing, cold compression, and topical anti-inflammation. Local cooling aids in vasoconstriction, theoretically limiting the solution dispersion, and it is recommended for the extravasation of hyperosmolar fluid [14]. The standard treatment protocol of PRRT includes the infusion of high-concentrated amino acid solution over 4 h starting 30 min before the administration of the radiolabeled-somatostatin analog. If the extravasation occurs during the infusion, we recommend continuing infusion via another IV access point.

The first patient continued to receive high-concentrated amino acid infusion during the third cycle of PRRT, and his follow-up serum creatinine level and eGRF (CKI-EPI) were unchanged. However, the second patient refused the infusion of high-concentrated amino acid solution during the fourth to sixth cycles of PRRT after experiencing extravasation. Her follow-up serum creatinine level was normal. Her eGFR (CKD-EPI) declined 2 months after the sixth cycle, but it remained within the normal range. This could probably be explained by the properties of Lu-177 and the patient’s baseline renal function. First, the beta-emitter Lu-177 has lower energy (147 keV) and a shorter tissue penetration range (2 mm) than Y-90, leading to lower renal radiation exposure and less tissue damage [2]. Second, the patient had normal baseline renal function. Svensson et al. found that patients with inferior renal function were exposed to a higher renal absorbed dose per administered activity [17].

Long-term nephrotoxicity resulting in decreased eGFR can occur after PRRT despite kidney protection if the kidneys are unable to regenerate their functional tissue after radiation-induced injury [18]. Regular and long-term evaluation of renal function was initially planned for this patient, but she died a few months after treatment.

## 4. Conclusions

The extravasation of lysine and arginine solution during PRRT can cause cutaneous complications. Despite the prevention of intravenous leakage, health care providers should be aware of the proper management of this complication once it occurs. Preserved renal function was demonstrated after [^177^Lu]Lu-DOTA-TATE treatment in the absence of the infusion of a high-concentrated amino acid solution to prevent kidney dysfunction. However, long-term follow-up of renal function is suggested.

## Data Availability

No new data were created or analyzed in this study. Data sharing is not applicable to this article.
